# Predatory Functional Response and Prey Choice Identify Predation Differences between Native/Invasive and Parasitised/Unparasitised Crayfish

**DOI:** 10.1371/journal.pone.0032229

**Published:** 2012-02-16

**Authors:** Neal R. Haddaway, Ruth H. Wilcox, Rachael E. A. Heptonstall, Hannah M. Griffiths, Robert J. G. Mortimer, Martin Christmas, Alison M. Dunn

**Affiliations:** 1 School of Environment, Natural Resources and Geography, College of Natural Sciences, Bangor University, Bangor, United Kingdom; 2 Institute of Integrative and Comparative Biology, Faculty of Biological Sciences, University of Leeds, Leeds, United Kingdom; 3 School of Earth and Environment, University of Leeds, Leeds, United Kingdom; 4 Environment Agency, Leeds, United Kingdom; University of Bristol, United Kingdom

## Abstract

**Background:**

Invasive predators may change the structure of invaded communities through predation and competition with native species. In Europe, the invasive signal crayfish *Pacifastacus leniusculus* is excluding the native white clawed crayfish *Austropotamobius pallipes*.

**Methodology and Principal Findings:**

This study compared the predatory functional responses and prey choice of native and invasive crayfish and measured impacts of parasitism on the predatory strength of the native species. Invasive crayfish showed a higher (>10%) prey (*Gammarus pulex*) intake rate than (size matched) natives, reflecting a shorter (16%) prey handling time. The native crayfish also showed greater selection for crustacean prey over molluscs and bloodworm, whereas the invasive species was a more generalist predator. *A. pallipes* parasitised by the microsporidian parasite *Thelohania contejeani* showed a 30% reduction in prey intake. We suggest that this results from parasite-induced muscle damage, and this is supported by a reduced (38%) attack rate and increased (30%) prey handling time.

**Conclusions and Significance:**

Our results indicate that the *per capita* (i.e., functional response) difference between the species may contribute to success of the invader and extinction of the native species, as well as decreased biodiversity and biomass in invaded rivers. In addition, the reduced predatory strength of parasitized natives may impair their competitive abilities, facilitating exclusion by the invader.

## Introduction

Biological invasions are one of the main causes of biodiversity loss and changes in community structure [1], [2], [3], [4]. Invasive predators may inflict stronger regulatory pressures on native prey populations then their native counterparts. For example, a meta-analysis of field experiments with mammalian and avian predators revealed that alien predators had an impact double that of native predators [5]. Invasive predators can change the structure of the invaded community through predation pressure on native prey as well as through competition with native predators [6], [7]. For example, successive invasion of a North American lake by lake trout and mysid shrimp (*Mysis diluviana*) predators caused a reduction in native predators and a trophic cascade affecting phytoplankton, zooplankton, fish, and a non-aquatic predator [8].

The signal crayfish, *Pacifastacus leniusculus* modifies native communities in Europe [9], [10], [11] through burrowing activities [12], [13] and trophic interactions with native species [14]. *P. leniusculus* introductions have been associated with reductions in overall invertebrate diversity and richness [15] and overall invertebrate biomass [16], [17]. In the UK, *P. leniusculus* is replacing the native crayfish *Austropotamobius pallipes* through competition for habitat and food, and through outbreaks of crayfish plague (caused by the fungus *Aphanomyces astaci*), for which *P. leniusculus* acts as a reservoir [18]. Studies of invaded communities indicate that *P. leniusculus* has a stronger impact on its prey species than does the native crayfish. For example, Peay *et al.* [14] observed a decrease in the abundance of juvenile trout (*Salmo* spp) following the replacement of native crayfish by *P. leniusculus* in Yorkshire. It is not clear whether these negative effects reflect the high densities attained by the invader present in many water bodies [19] or a higher *per capita* impact relative to the native *A. pallipes*.

Whilst potential invaders can be identified [20], elucidating the extent of their impacts is a greater challenge. Invading predators may differ from native predators in their prey choice and their impact on native prey species. A small number of authors have investigated the predatory functional response (the consumption of a prey by a predator in relation to the density of that prey [21]) in invasive species as a potential predictor of invader effects [22], [23], [24]. Modelling of predatory functional responses is typically carried out using one of three models; type I, type II and type III (see [21]). In a basic type I functional response prey consumption rises linearly with increasing prey density. The type II functional response features a deceleration in prey consumption with increasing prey density towards a plateau. Type III functional responses have a similar plateau to type II functional responses, but at low prey density there is an acceleration in prey consumption as prey density increases, forming an ‘S-shaped’ curve.

Bollache *et al.* [25], for example, found the Ponto-Caspian invasive amphipod *Dikerogammarus villosus* to possess a higher type II functional response than native *Gammarus* species, underlying changes in food webs in invaded rivers.

Native and invasive species may also differ in their choice of prey. By comparing hierarchies of choice in food items with different characteristics (such as mobility and defences) inferences can be made on the likely impacts of invasive relative to native predators [26].

Parasites can play critical roles in structuring biological communities and may mediate the success and impacts of biological invasions [27], [28], [29]. Parasites can influence predator-prey dynamics through density-dependent effects on the host. For example, competitive replacement of the European red squirrel *Sciurus vulgaris* by the grey squirrel *Sciurus carolinensis* is occurring 25 times more rapidly in the UK than in mainland Europe as a result of squirrel poxvirus [29]; the virus is lethal to red squirrels but is asymptomatic in greys which act as a reservoir for the disease. In the US, outbreaks of canine parvovirus in the grey wolf (*Canis lupus*) led to a crash in the wolf population and release from predator regulation of its moose (*Alces alces*) prey [30]. In addition to density-mediated effects, parasites can also mediate invader-native interactions through effects on host behaviour (trait mediated indirect effects, TMIEs; [31]), with knock-on effects on the community structure [28], [32]. For example, Dick *et al.* [33] recently demonstrated that infection with *Echinorhyncus truttae* (an acanthocephalan) led to an increase in the predatory strength of the invasive amphipod *Gammarus pulex* on the endemic invertebrate *Asellus aquaticus*.

The native European crayfish, *A. pallipes* is infected by the microsporidian parasite *Thelohania contejeani* [27], [34], [35]. Unlike crayfish plague, *T. contejeani* causes a chronic infection. The parasite infects muscle fibres, restricting movement, eventually leading to death [36]. We predict that *T. contejeani* will change the predatory impact of its host, reducing its prey intake and shifting prey choice towards those items with low capture and handling demands.

Here we compare the predatory functional response of the invasive crayfish (*P. leniusculus*) and native crayfish (*A. pallipes*) on the common prey *Gammarus pulex* (Amphipoda), and measure the impact of parasitism by *T. contejeani* on the predatory strength of *A. pallipes*. We also compare the prey choice of the invasive and native species, and investigate the impact of parasitism on prey choice.

## Methods

University of Leeds Research Ethics Committee ethical approval was not required since the work described herein did not include human participants or their data, genetically modified plant material, or have the potential to adversely affect the environment. Furthermore, no work involved regulated procedures under the UK Animals (Scientific Procedures) Act 1986. All necessary permits were obtained for the described field studies. Neal Haddaway held a current Natural England license for work with *A. pallipes* at the time of this work. Environment Agency trapping and removal licenses were obtained to collect crayfish from Bolton Abbey and Wyke Beck. No licenses were necessary for the collection of other animals.

### Experimental Animals

*P. leniusculus* were collected from Bolton Abbey in the Upper River Wharf, Yorkshire UK (NGR; SE071539, Lat/Long; 53.9809/-1.8917), that drains into the River Ouse. *A. pallipes* were collected from Wyke Beck, Yorkshire, in the Aire catchment (NGR; SE341364, Lat/Long; 53.8225/-1.4819). All animals were obtained in June 2009. Crayfish were size-matched in order to reduce the influence of size-related differences between groups. *P. leniusculus* grow faster than *A. pallipes* [37] and equally-sized animals of these species may potentially differ in age. However, the (adult) size range (30–35 mm) used in this experiment has already undergone major ontogenic shifts in diet as juveniles [38], hence any between species differences in predatory strength or prey choice are unlikely to result from age differences. Similarly, although parasites may lead to a reduction in growth [39], *T. contejeani*-infected *A. pallipes* have been found to live for only 1 or 2 years following infection [34]. As a result, growth in infected adults of the size used in our study is likely to have been minimally affected by the parasite, since infection would have occurred subsequent to attaining adult size.

Infection status of *A. pallipes* was assessed visually, based on the presence of opaque tail musculature [40]. Although some subclinical infections may be missed by this method [35], our experiments will test for differences in predation caused by patent infection. Previous screening of this population of *A. pallipes* has identified no other diseases (CEFAS, unpublished data or CEFAS pers com). None of the *P. leniusculus* were visibly infected by *T. contejeani*, and visible infection has not been reported in the literature. *T. contejeani* has recently been identified in *P. leniusculus* using molecular diagnosis, but was found to be asymptomatic [27]. Prior to experiments, crayfish were starved for 24 hours. Crayfish were held at the University of Leeds in constant environmental conditions; 16∶8 light∶dark regime at 17°C.

### Food Items

Amphipods, isopods, snails, pond-weed (*Chara sp.*) and dead leaves (common food items for crayfish [41]) were sourced from Meanwood Beck (NGR; 53°52′18″N 1°37′17″W). Bloodworm were sourced from a pet retailer. All prey animals were also found at source locations for *A. pallipes* and *P. leniusculus* (pers. obs.) and were thus previously experienced by the predators. Prey organisms were held at the University of Leeds in constant environmental conditions; 16∶8 light∶dark regime at 17°C. Fish (freshwater brown trout) was purchased from a grocery retailer in Leeds and frozen until use. Dead crayfish were defrosted from frozen samples of natural mortalities of a laboratory population. Sycamore leaves were collected in autumn and rotted in water for a minimum of two months prior to use.

### Prey Choice Experiment

#### Experimental Design

In order to compare prey choice, four treatment groups were used in all trials; *P. leniusculus*, healthy *A. pallipes*, *A. pallipes* with *T. contejeani*, and control (no crayfish). Ten replicates of one test crayfish were used per trial per crayfish group, and each crayfish was used twice; once for mobile and once for non-mobile trials.

Two sets of trials were undertaken. The first compared choice of the different mobile food items (amphipod, *Gammarus pulex*; isopod, *Asellus aquaticus*; snail, *Potamopyrgus jenkinsii*; bloodworm, unidentified chironomid larvae), the second compared choice of non-mobile items (live pond-weed, *Chara sp.*; decaying sycamore leaves, *Acer pseudoplatanus*; dead fish, *Salmo trutta*; dead crayfish, *A. pallipes*). In each case individual crayfish were given fixed masses of each of four items, either mobile or non-mobile. Food items were chosen because they covered a wide range of food types fed upon by crayfish.

Based on preliminary trials, individual crayfish were placed into 8 litre tanks containing 2 litres of dechlorinated tap water (approximately 5 cm deep) and one plastic shelter 5 cm in diameter. Each tank was then given a fixed mass of prey/food items (0.3 g of each food item) and left for 23 hours. At the end of this period crayfish were removed from their tanks and remaining prey was collected and weighed. Trials were carried out over five days, with two trials in each group per day.

#### Statistical Methods

Size matching between groups of crayfish was confirmed by comparing carapace lengths in R [42] using a linear model. Crayfish groups did not differ in carapace length (ANOVA; F = 1.80 df = 2,25 p = 0.186).

Total consumption was compared in R between groups using i) a generalised linear model (GLM) for mobile prey with quasipoisson error distribution since errors were not Normally distributed (Shapiro-Wilk; W = 0.913 p = 0.005), and ii) a linear model for non-mobile prey since errors were Normally distributed (Shapiro-Wilk; W = 0.968 p = 0.300). Accordingly, pairwise comparisons were modelled using GLMs with quasipoisson error distribution for mobile and linear models for non-mobile prey. No post-hoc correction was carried out on the resultant p-values, but results were considered with respect to both classical and Bonferroni-adjusted levels of α (0.05 and 0.017 respectively) [43].

Prey choice hierarchies were compared in R between groups using GLM for both mobile and non-mobile prey with quasibinomial error distributions (using the bound columns; ‘amount eaten’ and ‘amount remaining’ as the dependent variable), since both mobile and non-mobile data were over-dispersed (Dispersion Parameter = 75.46 and 82.10 respectively). Data for treatment groups was subject to correction for prey depletion during trials: mean reduction for each prey item from control trials was subtracted from each data point prior to analysis. Pairwise comparisons were modelled using GLMs with quasibinomial error distribution. In all cases where significant differences between predator cues were detected, pairwise comparisons were performed without adjustment of p-values [43]. Instead, Bonferroni adjustment of alpha (typically α = 0.05) was employed for clarity.

### Predatory Functional Response Experiment

#### Experimental Design

To test for differences in the predatory functional response between crayfish, three treatment groups were used; *P. leniusculus*, healthy *A. pallipes*, and *A. pallipes* infected with *T. contejeani*. Individual crayfish were supplied with *G. pulex* at 14 different prey densities (4, 6, 8, 10, 16, 20, 30, 40, 80, 130, 160, 220, 270, and 320) with eight replicates at each density within each treatment. The number of prey remaining after 24 hours was then measured. Experiments were run in 8 L tanks containing 2 litres of dechlorinated tap water (approximately 5 cm deep). Tank sides were covered in black plastic, and each animal was provided with one shelter (12 cm length of black plastic tubing 5 cm in diameter) in order to minimise stress. Crayfish were used only once within each prey density and each animal was used a maximum of 14 times. All were starved for 24 hours before each trial began. Trials were randomised through time, with at least 2 days' recovery time allowed between each trial.

#### Statistical methods

Size matching between crayfish groups was confirmed by comparing carapace lengths in R using a linear mixed effects model (LME) [44] with crayfish ID as a random factor and using a quasipoisson error distribution. Crayfish groups did not differ in carapace length (LMER; Chi-sq = 0.778 df = 2,332 p = 0.678).

Differences in overall prey consumption were assessed in R using a GLM with poisson error distribution. It is very difficult to differentiate between type II and type III functional responses due to the high variability inherent in such data [45]. In order to assess whether data conformed to type II or type III curves, therefore, proportional mortality was plotted against the number of prey supplied. Type II functional responses are characterised by significantly higher proportional mortality at low prey density than high prey density, whereas type III functional responses are characterised by significantly lower proportional mortality at low prey densities than high prey densities [46]. Discrimination between type I and type II responses has previously been carried out by comparing proportional mortality at different prey densities [47]. In this way, proportional mortality was tested using a GLM with a quasibinomial error distribution, which confirmed the presence of type II responses for all species (GLM; Residual Deviance = 1784.4 df = 1 p<0.001).

We compared the fit of two equations that describe type II functional responses. Firstly, Holling's [48] disc equation (adapted from [49]) describes a type II relationship (Equation 1).
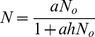
(1)where *N* is number of prey eaten, *a* is attack rate, *h* is handling time, and *N_o_* is number of prey supplied.

The encounter rate of prey by a predator declines as prey are eaten. The Rogers random-predator equation [50], [51] also describes a type II Functional Response but accounts for prey depletion (Equation 2).
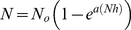
(2)Equation 2 must be modified since *N* is on both left and right sides of the equation. This has been done using the *Lambert W* function (W in Equation 3 below) by Bolker [52].
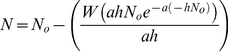
(3)Data were, therefore, modelled using both Holling type II and Rogers functional response curves using non-linear least squares regression (*nls*) for Holling type II functions, the packages emdbook for the Lambert W function (*lambertW*) [53] and bbmle for maximum likelihood estimation (*mle2*) [54]. Holling and Rogers curves were compared using Akaike Information Criterion (AIC) values to find the models of best fit (lower AIC implies a better model fit). The coefficients *a* and *h* were obtained and compared between predator groups using t-tests.

## Results

### Prey Choice

**Figure 1:**
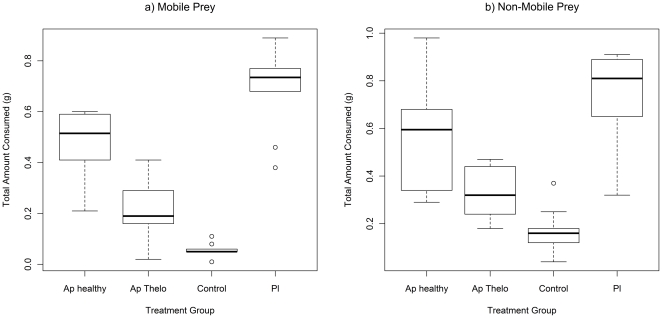
Total food consumption for crayfish. **a) mobile and b) non-mobile food items for** ***P. leniusculus*** **(Pl), healthy** ***A. pallipes*** **(Ap healthy),** ***A. pallipes*** **with** ***T. contejeani*** **(Ap Thelo), and controls.** Plots show medians (thick line), interquartile ranges (boxes) and data range (whiskers), with outliers as open circles.

Crayfish groups differed significantly in total food consumption for both mobile (GLM; Deviance = 7.389 df = 3,36 p<0.001) and non-mobile (ANOVA; F = 25.905 df = 3,36 p<0.001) prey over the experimental period (see Figure 1). For both mobile and non-mobile food items, *P. leniusculus* consumed more prey than *A. pallipes*, and *T. contejeani* infection significantly reduced prey intake by *A. pallipes*. Control treatments had minimal reduction in food mass, confirming that predation was the major factor responsible for the differences observed. Table 1 and Table 2 display the results of pairwise comparisons between crayfish groups for mobile and non-mobile prey. Significant differences were observed between all groups with the exception of *P. leniusculus* and healthy *A. pallipes* for non-mobile prey (ANOVA; F = 4.324 df = 1,18 p = 0.053 (unadjusted)).

**Table 1:** Pairwise linear model comparisons between crayfish groups in mobile prey consumption.

Comparison	Deviance	df	p
*P. leniusculus* -vs- healthy *A. pallipes*	0.413	1,18	0.001*
*P. leniusculus* -vs- *A. pallipes* with *T. contejeani*	2.681	1,18	<0.001*
Healthy *A. pallipes* -vs- *A. pallipes* with *T. contejeani*	1.020	1,18	<0.001*
*P. leniusculus* -vs- control	6.340	1,18	<0.001*
*A. pallipes* with *T. contejeani* -vs- control	3.725	1,18	<0.001*
Healthy *A. pallipes* -vs- control	0.950	1,18	<0.001*

P-values are uncorrected for multiplicity, and are instead reported with Bonferroni adjustment of α from 0.05 to 0.017;

* denotes significance at the Bonferroni adjusted level of α. Deviance reported for Analysis of Deviance (generalised linear models), F-statistic reported for ANOVA (linear models).

**Table 2:** Pairwise linear model comparisons between crayfish groups in non-mobile prey consumption.

Comparison	F	df	p
*P. leniusculus* -vs- healthy *A. pallipes*	4.324	1,18	0.053
*P. leniusculus* -vs- *A. pallipes* with *T. contejeani*	37.942	1,18	<0.001*
Healthy *A. pallipes* -vs- *A. pallipes* with *T. contejeani*	9.242	1,18	<0.007*
*P. leniusculus* -vs- control	78.037	1,18	<0.001*
*A. pallipes* with *T. contejeani* -vs- control	28.342	1,18	<0.001*
Healthy *A. pallipes* -vs- control	14.044	1,18	<0.001*

P-values are uncorrected for multiplicity, and are instead reported with Bonferroni adjustment of α from 0.05 to 0.017;

* denotes significance at the Bonferroni adjusted level of α. Deviance reported for Analysis of Deviance (generalised linear models), F-statistic reported for ANOVA (linear models).

**Figure 2:**
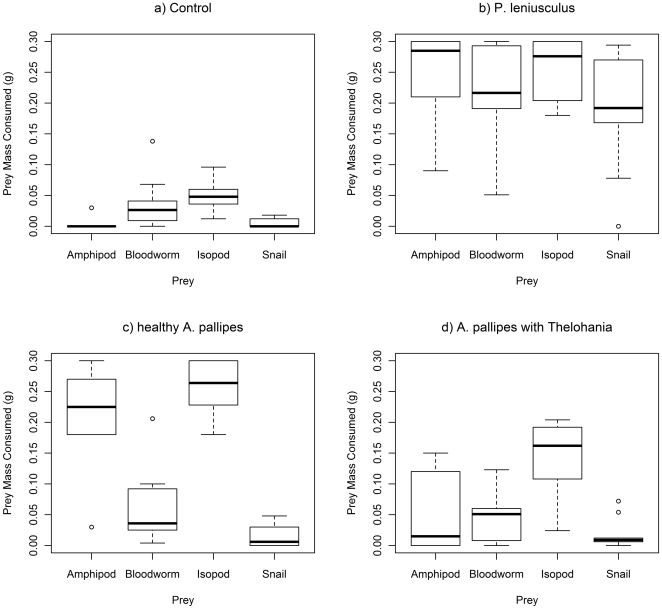
Prey consumption (g) by crayfish predators for mobile food items. Trials with *P. leniusculus*, healthy *A. pallipes*, *A. pallipes* with *T. contejeani*, and controls. Plots show medians (thick line), interquartile ranges (boxes) and data range (whiskers), with outliers as open circles.

**Figure 3:**
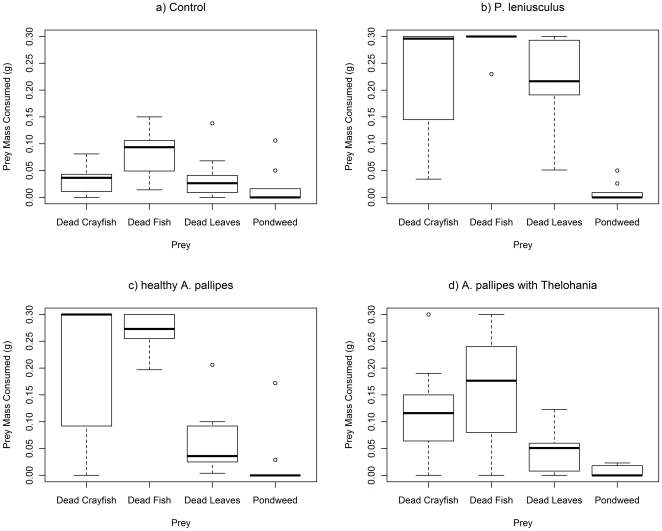
Prey consumption (g) by crayfish for non-mobile food items. Trials with *P. leniusculus*, healthy *A. pallipes*, *A. pallipes* with *T. contejeani*, and controls. Plots show medians (thick line), interquartile ranges (boxes) and data range (whiskers), with outliers as open circles.

Figure 2 and 3 show the mass of prey consumed for mobile and non-mobile food items respectively. The presence of a significant interaction between group and prey item indicated that groups differed in their prey choice hierarchy for both mobile (GLM; Deviance = 2.102 df = 6,108 p<0.001) and non-mobile (GLM; Deviance = 1.241 df = 6,108 p = 0.017) food. For mobile prey, pairwise comparisons indicated that the difference lay between healthy *A. pallipes* and *P. leniusculus* (GLM; Deviance = 1.785 df = 3,72 p<0.001), with marginal differences (significant at α = 0.05 but not at Bonferroni α = 0.017) between the other two pairs (see Table 3). *P. leniusculus* consumed all four mobile prey items in similar amounts, whilst *A. pallipes* consumed prey in the following hierarchy: healthy – isopods>amphipods>bloodworm>snail; *T. contejeani*-infected – isopods>bloodworm>amphipods>snail.

**Table 3:** Pairwise generalised linear model comparisons between crayfish groups in prey choice for mobile food items.

Comparison	Deviance	df	p
*P. leniusculus* -vs- healthy *A. pallipes*	1.785	3,72	<0.001*
*P. leniusculus* -vs- *A. pallipes* with *T. contejeani*	0.568	3,72	0.042
Healthy *A. pallipes* -vs- *A. pallipes* with *T. contejeani*	0.524	3,72	0.042

Unadjusted P-values have been uncorrected for multiplicity, and are instead reported with Bonferroni adjustment of α from 0.05 to 0.017;

* denotes significance at the Bonferroni adjusted level of α.

For non-mobile prey, pairwise comparisons indicated that *P. leniusculus* and healthy *A. pallipes* differed significantly (GLM; Deviance = 1.192 df = 3,72 p = 0.004), but that other groups did not differ (see Table 4). Non-mobile food items were consumed in similar patterns by *P. leniusculus* and *A. pallipes* (dead fish>dead crayfish>dead leaves>pond-weed), although *A. pallipes* consumed less dead leaves than *P. leniusculus*. Less overall consumption of non-mobile food was evident in *T. contejeani*-infected *A. pallipes*, although the prey choice hierarchy did not differ.

**Table 4:** Pairwise generalised linear model comparisons between crayfish groups in prey choice for non-mobile food items.

Comparison	Deviance	df	p
*P. leniusculus* -vs- healthy *A. pallipes*	1.192	3,72	0.004*
*P. leniusculus* -vs- *A. pallipes* with *T. contejeani*	0.289	3,72	0.212
Healthy *A. pallipes* -vs- *A. pallipes* with *T. contejeani*	0.135	3,72	0.669

Unadjusted P-values have been uncorrected for multiplicity, and are instead reported with Bonferroni adjustment of α from 0.05 to 0.017;

* denotes significance at the Bonferroni adjusted level of α.

### Predatory Functional Responses

**Figure 4:**
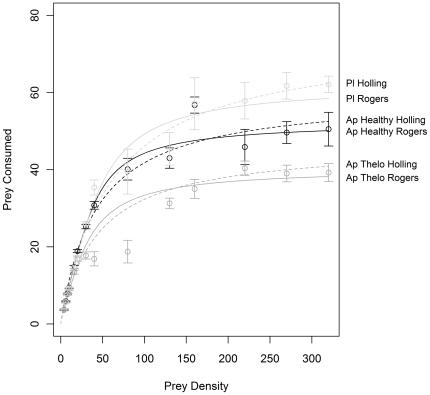
Holling type II (dashed lines) and Rogers random-predator (solid lines) functional response curves for crayfish. Healthy *A. pallipes* (Ap healthy), *A. pallipes* with *T. contejeani* (Ap thelo), and *P. leniusculus* (Pl) at different densities of *Gammarus pulex*. Circles represent mean number of prey consumed and vertical bars represent one standard error.

The predatory functional response curves of both healthy *A. pallipes* and *A. pallipes* with *T. contejeani* were lower than that of *P. leniusculus*, whilst *A. pallipes* infected with *T. contejeani* also demonstrated a lower curve than that of apparently healthy conspecifics (Figure 4). All three crayfish groups have reached asymptotes within the prey densities supplied in this investigation. Initial examination of the curves using a general linear model showed that the significance of an interaction between prey density supplied and crayfish group (GLM: Residual Deviance = 323.0 df = 26,294 p = 0.002) indicates that some of the groups differed in their functional responses (see Table 5). Using a Bonferroni adjusted alpha (0.017) there is a significant difference between *P. leniusculus* and *A. pallipes* with *T. contejeani*. The other pairwise comparisons, however, show marginal p-values that warrant further investigation using the Holling type II and the Rogers random-predator equations that follow.

**Table 5:** Pairwise generalised linear model comparisons between crayfish groups in overall shape of functional response.

Comparison	Residual Deviance	df	p
*P. leniusculus* -vs- healthy *A. pallipes*	83.3	13,84	0.08
*P. leniusculus* -vs- *A. pallipes* with *T. contejeani*	98.74	13,84	0.01
Healthy *A. pallipes* -vs- *A. pallipes* with *T. contejeani*	55.17	13,84	0.05

P-values have been uncorrected for multiplicity, and are instead reported with Bonferroni adjustment of α from 0.05 to 0.017.

The fit of the two models was compared; the Holling type II equation that does not account for prey depletion, and the Rogers random-predator equation that does account for prey depletion. AIC values for functional response curves for each predator group are given in Table 6. Lower values were obtained for Rogers random-predator functions for all crayfish groups, indicating that accounting for prey depletion resulted in models of better fit. Since Rogers random-predator functions were a better fit for the data, further analysis was based on coefficients from the Rogers functions.

**Table 6:** Akaike Information Criterion (AIC) for two functional response models fitted for three crayfish groups.

Model	*A. pallipes* healthy	*A. pallipes* with *T. contejeani*	*P. leniusculus*
Rogers Random-Predator	553.9	616.2	781.0
Holling Type II	749.0	668.4	810.8

A lower AIC indicates a better fit.

Using the parameters derived from the Rogers random-predator equation, *P. leniusculus* and healthy *A. pallipes* did not differ significantly in attack rate (Table 7) (t-test; t = 1.87 df = 1 p = 0.062) (although there was a trend towards a greater attack rate by the native species), whereas *T. contejeani*-infected *A. pallipes* had a lower attack rate than both *P. leniusculus* (t-test; t = 4.01 df = 1 p<0.001) and healthy *A. pallipes* (t-test; t = 5.45 df = 1 p<0.001). All three crayfish groups differed significantly in handling time; *P. leniusculus* had a lower handling time than *T. contejeani*-infected *A. pallipes* (t-test; t = 11.35 df = 1 p<0.001) and healthy *A. pallipes* (t-test; t = 5.58 df = 1 p<0.001), and healthy *A. pallipes* had a lower handling time than did *T. contejeani*-infected *A. pallipes* (t-test; t = 7.11 df = 1 p<0.001).

**Table 7:** Rogers random-predator attack rates and handling times for crayfish functional responses.

	Rogers Random-Predator Function			
	Attack rate (a)	SE	Handling time (h)	SE
*P. leniusculus*	3.210	0.167	1.602×10^−2^	3.444×10^−4^
*A. pallipes* (healthy)	3.711	0.209	1.903×10^−2^	4.156×10^−4^
*A. pallipes* (*T. contejeani*)	2.318	0.147	2.473×10^−2^	6.859×10^−4^

Predicted from functional response models of healthy *A. pallipes*, *A. pallipes* with *T. contejeani*, and *P. leniusculus* at different densities of *Gammarus pulex*. SE; standard error.

## Discussion

The Invasive crayfish *P. leniusculus* displayed a greater overall predatory strength than did the native crayfish *A. pallipes*, and showed less ‘choosiness’ for mobile invertebrates relative to native crayfish. The observed lack of choosiness by *P. leniusculus* is in accord with studies by Gherardi and Barberesi [55]. The invasive species consumed 83% more prey overall than did its native competitor when offered a range of food items (Figure 1). The invader also preyed at a 10% higher rate in the predatory functional response experiment, probably reflecting a shorter (by 16%) prey handling time in comparison with the native species. Similarly, the invasive crayfish *Procambarus clarkii* was found to display shorter handling times than the native *A. italicus* in Italy [56]. Interestingly however, a study of *P. leniusculus* in its native range showed that it handled and consumed snails faster than did the invasive *P. clarkii* and *Orconectes virilis* [57].

Our results are in accord with predictions of higher functional responses in damaging invaders than their native counterparts [22]. *P. leniusculus* has rapidly invaded European waters since its introduction for aquaculture in the 1960s [58], causing detrimental impacts on recipient communities and ecosystems [11], [12], [14], [59], [60] including extinction of the native *A. pallipes* across large areas [18], [58]. The *per capita* difference observed here in the predatory impact of the invasive versus the native crayfish is likely to contribute to success of the invader and its impact on the recipient community.

In the wild, the differences between the predatory impact of the native and invasive crayfish are likely to be greater than that observed here as a result of differences in the size and densities of the species. The differences in predatory strength observed in the current study may be conservative as crayfish size was controlled in these experiments; in the wild, *P. leniusculus* shows a faster growth rate and reaches larger adult size [37] than the native species. Furthermore, the invader has also been found to reach higher densities in the field [61] hence the differences between the species' predatory impacts in the wild are likely to result from differences in both functional response and numerical response.

Parasites play important roles in ecosystem functioning by influencing species coexistence patterns, energy flow and community stability [28], [62], [63]. The density-mediated effects of parasites may regulate host populations [64], and hence mediate biological invasions [29], [65], [66]. There is also increasing realisation of the importance of the sublethal effects of parasites (effects on host morphology and behaviour) in mediating trophic interactions with other members of the community and in changing invasion impacts [28], [63], [67], [68]. In comparison with density-mediated effects, these trait-mediated effects can operate on shorter timescales, influencing community structure at a faster rate [31]. Parasitism had a significant effect on predation by the native *A. pallipes*; infected individuals ate 55% less mobile prey and 41% less non-mobile food. *T. contejeani* causes a chronic infection in its host, affecting the muscle tissue and leading to reduced motility and eventual death. The observed reduction in the predatory strength of *A. pallipes* may therefore reflect reduced muscle function in the host and a lower metabolic rate in infected hosts. The large reduction in predation of mobile items is in accord with our prediction that the parasite should cause a shift towards prey with lower capture and handling demands. Also in accord with this prediction, infected *A. pallipes* showed a 22% reduction in the intake of *G. pulex* in the functional response experiment reflecting a 38% reduction in attack rate and an increase (30%) in handling time. The observed reduction in predatory strength in crayfish infected with *T. contejeani* contrasts with an increase in predatory strength found in the invasive *Gammarus pulex* infected by the acanthocephalan parasite *Echinorhynchus truttae* [33].

The reduction in predatory strength of infected *A. pallipes* is likely to affect both its predatory and competitive interactions. Prevalence of *T. contejeani* varies; whilst prevalence is below 10% in many *A. pallipes* populations [34], recent studies have revealed prevalences up to 50% in UK rivers [35]. By modifying the predatory strength of the native crayfish *A. pallipes*, *T. contejeani* may reduce the impact of this predator on its macroinvertebrate prey. Furthermore, the parasite-induced reduction in predatory strength may facilitate competitive exclusion of the host by the invasive signal crayfish *P. leniusculus*, with ramifications throughout the lower trophic levels in the community.

Invasive species often achieve higher densities than their native competitors [7], [69] and hence have greater predatory and competitive impacts. Our results indicate that a *per capita* (i.e. functional response) difference between the species may also contribute to success of an invader and its impact on the recipient community. In addition, the reduced predatory strength of parasitized natives may reduce their competitive abilities, facilitating exclusion by the invader. Understanding and predicting the consequences of biological invasions will be enhanced by further study of *per capita* differences in predatory impact and of parasite-induced modification of predatory behaviour.
